# Associations between Psychosocial and Physiological Factors and Diabetes Health Indicators in Asian and Pacific Islander Adults with Type 2 Diabetes

**DOI:** 10.1155/2013/703520

**Published:** 2013-10-24

**Authors:** Dongmei Li, Jillian Inouye, Jim Davis, Richard F. Arakaki

**Affiliations:** ^1^Department of Public Health Sciences, University of Hawaii, Honolulu, HI 96822, USA; ^2^Schools of Nursing and Allied Health Sciences, University of Nevada, Las Vegas, NV 89154, USA; ^3^John A. Burns School of Medicine, University of Hawaii, Honolulu, HI 96813, USA

## Abstract

The associations between psychosocial and physiological factors and diabetes' health indicators have not been widely investigated among Asians and Pacific Islanders. We hypothesize that health behaviour and depression are directly or indirectly associated with diabetes' health indicators such as BMI, glycemic control, general health, and diabetes quality of life. Our hypothesis was tested through a structural equation modelling (SEM) approach. Questionnaires that assessed health behaviour, depression, general health, diabetes quality of life, and haemoglobin A1c (HbA1c), along with patients' demographic information, were obtained from 207 Asian and Pacific Islander adults with type 2 diabetes. IBM SPSS Amos 20 was used for the SEM analysis at 5% level of significance, and the goodness fit of the SEM model was also evaluated. The final SEM model showed that diet and exercise and foot care had positive associations, while depression had a negative association with diabetes' health indicators. The results highlighted the importance of exercise and depression in diabetes patients' BMI, glycemic control, general health, and quality of life, which provide evidence for the need to alleviate patients' depression besides education and training in diet and exercise in future intervention studies among Asians and Pacific Islanders with type 2 diabetes.

## 1. Introduction

Diabetes is a chronic disease that affects about 11.3% of all people aged 20 years and older in the USA [1] and a growing epidemic within ethnic minority populations including Pacific Islanders and Asian Americans [2]. Health behaviours such as diet, exercise, and depression contribute to diabetes' health indicators such as BMI, glycemic control, quality of life, and general well-being. Previous studies demonstrated the direct association between health behavior and glycemic control [3–6]. A current study also found that physical exercise can control, prevent, or delay the onset of type 2 diabetes by markedly improving the low insulin sensitivity in at-risk patients [7]. A recent intervention programme conducted in Australia revealed the negative association between exercise and depression [8]. They found that overweight participants with heart disease and diabetes were less likely to report depressive symptoms after adjusting for treatment group and weight change, if they met recommendations for total duration of exercise. An important relationship between depressive symptoms and a heightened metabolic risk for type 2 diabetes, including prediabetes and impairment of *β*-cell function relative to insulin sensitivity, was observed in obese adolescents by a recent study conducted in the USA [9]. Other recent studies in Brazil and the USA also indicated the significant association between physical activity, depression, and diabetes patients' general health and quality of life [10, 11].

The social cognitive model [12, 13] pointed to the association between health behaviour, depression, and health outcomes. Many studies in the mainland USA have identified multiple factors that affect diabetes intervention in separate Asian groups [14–16]. The associations between the psychosocial and physiological factors and health outcomes have not been prospectively investigated in Asian and Pacific Islander (API) populations with type 2 diabetes. In this study, we use a structural equation modelling approach to examine the association between health behaviour, depression, and health outcome indicators such as BMI, glycosylated haemoglobin (HbA1c), quality of life, and general health in 207 Asian and Pacific Islander adults with type 2 diabetes who were recruited through a diabetes intervention study in Hawaii.

## 2. Materials and Methods

### 2.1. Data Source

The psychosocial, physiological, and health outcome data were obtained at baseline from API participants with type 2 diabetes enrolled in a randomized clinical trial of a cognitive behavior intervention to enhance diabetes self-management (NCT01182701). The clinical trial was conducted in compliance with the Helsinki Declaration and approved by the Committee on Human Studies of the University of Hawaii-Manoa (CHS #12473), and all participants signed informed consent prior to entry into this study. Two hundred and seven Asians and Pacific Islanders were between the ages of 18 and 75 years old and were trained in diabetes self-management such as performing self-monitoring of blood glucose (SMBG), keeping daily records, and understanding food and calorie concepts. Subjects with physical difficulties and diabetic complications such as severe eye disease, chronic renal failure, peritoneal dialysis, transplant recipient, foot amputation, congestive heart failure, stroke, and other conditions that limit activities were excluded from the study.

### 2.2. Variables

The following health and clinical measures were used in the structural equation modelling (SEM) analysis.

#### 2.2.1. Diabetes Quality of Life (DQOL)

This questionnaire is a 46-item multiple-choice assessment for adolescents and adults with insulin-dependent diabetes mellitus [17]. The subscales that were related to type 1 diabetes were omitted from this questionnaire. Satisfaction with quality of life, impact of diabetes, diabetes worry, and social/vocational worry are rated from 1 (very satisfied or no impact/no worry) to 5 (very dissatisfied or very impacted/worried). Convergent validity was established using the symptom checklist for the diabetes worry and social/vocational worry scales; the affect balance scale for correlation with global satisfaction; and the psychosocial adjustment to illness scale for correlation with the impact scale. The internal consistency of the DQOL measure in the Asian and Native Hawaiian and other Pacific Islander (NHOPI) groups based on Cronbach's alpha was good at 0.78. Principal component analysis was conducted to determine the number of factors in the DQOL scale, and only one factor was extracted, so the subscale was summarized to form the DQOL scores. The DQOL scores range from 0 to 100, with higher scores indicating better quality of life.

#### 2.2.2. General Health

A subscale of the medical outcome study of a 36-item short-form health survey (SF-36) assesses self-appraised general health. It consists of five items (Cronbach's alpha = 0.78) rated on a five-point scale. Results from a principal component analysis showed only one component. Thus, all items were summarized to form the SF-36 scores. The SF-36 was designed to measure functioning and well-being in people age 14 years and older [18]. The SF-36 scores have a range from 0 to 100, with higher scores defining a more favourable health state.

#### 2.2.3. Depression

 Depression assessment utilized the Center for Epidemiologic Studies-Depression (CES-D) scale, a 20-item self-report scale designed to measure current depressive symptomatology including depressed mood, feelings of guilt and worthlessness, helplessness and hopelessness, psychomotor retardation, loss of appetite, and sleep disturbance [19]. The CES-D has been used with Native Hawaiian populations, and its validity supported its use as an appropriate tool to screen for depression among adolescents of Native Hawaiian and other minority backgrounds [20, 21]. The internal consistency of the CES-D scale in our NHOPI population is satisfactory at 0.82, according to Cronbach's alpha. Principal component analysis showed only one component for the CES-D scale. All items were summarized to form the CES-D scores. The CES-D scores have a range between 0 and 60. High scores on the CES-D indicate high levels of distress. A CES-D score of ≥16 suggests a clinically significant level of psychological distress.

#### 2.2.4. Health Behaviour

The Summary of Diabetes Self-Care Activities (SDSCA) questionnaire is a self-report measure of the frequency of completing different self-care activities over the preceding seven days [22]. These activities include diet, exercise and footcare, glucose testing, and medication taking in a 12-item instrument (Cronbach's alpha = 0.69). Three subscales were formed according to the principle component analysis: (1) diet, (2) exercise and foot care, and (3) glucose testing and medication taking. All three subscales have scores ranging and from 0 to 7, with higher values indicating better self-care activities.

#### 2.2.5. Glycemic Control

Hemoglobin A1c (HbA1c) is the most widely used measure for glycemic control that measures the three-month average blood glucose concentrations; thus, it is not affected by the blood glucose level at a single time point. The participants' HbA1c levels were obtained from local clinical laboratories, with higher HbA1c values indicating worse glycemic control. For patients with type 2 diabetes, a good glycemic control has HbA1c levels at or below 6.5–7.0%.

### 2.3. Statistical Analysis

Summary statistics and frequency distributions were calculated to illustrate the demographic and clinical characteristics of the 207 Asian and Pacific Islander participants. Pearson's correlation coefficients were used to assess the correlation between the psychological and physiological factors and diabetes' health indicators. SEM was then used to develop an association model between psychological and physiological factors and diabetes' health indicators based on previous studies on the association between those variables and Pearson's correlation coefficient matrix.

SEM is a statistical technique used to estimate and test a theoretical framework with linear-related observed or unobserved variables. It uses the correlation or covariance matrix among the variables rather than the raw data as the input format to test the validity of the theoretical model based on the assumption that the population correlation or covariance matrix will be reproducible by SEM if the theoretical model is correct and the parameters are known.

The goodness fit of the SEM models is evaluated by chi-square test, root mean square error of approximation (RMSEA), and comparative fit index (CFI). The chi-square test measures the difference between the observed correlation or covariance matrix and the model correlation or covariance matrix. A chi-square test with *P  *value greater than 0.05 indicates a good fit. RMSEA estimates the lack of fit in the current model compared to a saturated model. A good model is considered to have a RMSEA of .05 or less. Models whose RMSEA is 0.1 or more have a poor fit. CFI examines the fit of current model relative to independence models. A good model will have CFI values greater than 0.90. The SEM analysis was conducted using the IBM SPSS Amos 20 software.

### 2.4. Social Cognitive Model

The social cognitive model [13, 14] posits that one's emotions and behaviors will lessen his/her perceived difficulty in healthy lifestyle change and improve his/her health eventually. Based on the social cognitive model, we propose that health behavior (SDSCA) and depression will affect health outcome indicators such as BMI, glycemic control (HbA1c), depression, diabetes quality of life, and general health. 

We tested the social cognitive model on psychosocial, physiological, and health indicators of the 207 Asians and Pacific Islanders, using the SEM approach. All the parameters in the SEM model were estimated by a maximum likelihood method. We examined the association between diet, exercise and foot care, depression, BMI, HbA1c, general health, and diabetes quality of life.

## 3. Results

### 3.1. Demographic and Clinical Characteristics

The baseline characteristics of the 207 participants revealed an average age of 57.4 years with a slightly higher proportion of females (54.6%) than males (45.4%) (Table 1). The majority of the participants were married (69.8%) and nonsmokers (90.0%) and had some college or higher degree education (79.5%). More than half of the participants perform professional or technical work (52.6%) and have income over $45,000/year (61.2%). The Japanese had the highest prevalence (36.2%), followed by Hawaiian and Pacific Islanders (25.6%), other Asians (17.4%; Chinese, Koreans, and others), Filipino (14.0%), and mixed races (6.8%). 

Table 1 also showed the clinical measurements of the 207 participants. The majority of the Asian and Pacific Islander participants (89.2%) were overweight or obese with mean BMI of 32.2. Most participants (70.1%) did not have good glycemic control (HbA1c ≥ 7%), as the mean level of HbA1c is 8.0. Eighteen percent of participants were depressed with mean depression scores of 10.1. The mean general health score was 69.6, and the mean diabetes quality of life score was 73.6. For self-care activities, the participants had a mean of 3.6 for diet, 3.2 for exercise and foot care, and 4.7 for blood glucose and medication taking.

### 3.2. Pearson's Correlation Matrix

Pearson's correlation matrix showed the pairwise association, between psychosocial and physiological variables and health indicators (Table 2). BMI had moderately negative associations with exercise and foot care, general health, and diabetes quality of life. Higher BMI was associated with elevated HbA1c levels. Diet had a weak positive association with exercise and foot care and diabetes quality of life. Meanwhile, diet had a weak negative association with depression. Exercise and foot care has a weak positive association with general health. Blood glucose and medication taking did not seem to be correlated with any of the other variables. Depression showed a strong negative correlation with general health and a moderately negative association with diabetes quality of life. Higher HbA1c levels were moderately associated with lower general health and lower diabetes quality of life. Better general health was strongly associated with better quality of life.

### 3.3. SEM Model

**Figure 1:**
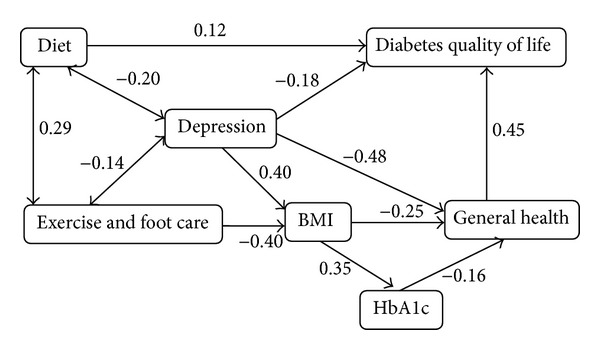
Final SEM with standardized coefficient weights on the associations between psychosocial and physiological factors and diabetes health indicators in Asian and Pacific Islander adults with type 2 diabetes.

Based on the Pearson correlation matrix and previous studies on psychosocial and physiological effects on health indicators, SEM was used to study the association between health behavior, depression, and health indicators of BMI, HbA1c, general health, and diabetes quality of life. Correlations were used for associations where theory did not strongly suggest the direction of effect. Blood glucose and medication were excluded from the SEM model due to a lack of correlation with all other variables.  Figure 1 showed the fitted SEM with estimated standardized regression coefficients and correlations. To provide a common unit for comparison of the various paths, regression results are presented as standardized coefficients giving associations in standard deviation units. More complete regression results are provided in Table 3, including unstandardized regression coefficients that give associations in the original measurement scales. The estimated correlations between diet, depression, and exercise and foot care were shown in Table 4. 

The standardized total effect, direct effect, and indirect effect were summarized in Table 5. The total effect can be decomposed into direct effect and indirect effect. For example, the total effect of depression on general health (−0.60) included the direct effect of depression (−0.48) and the indirect effect of depression (−0.12). The indirect effect of depression (−0.12) included the indirect path through BMI (0.40 ∗ −0.25 = −0.10) and the indirect path through both BMI and HbA1c (0.40 ∗ 0.35 ∗ −0.16 = −0.02). Depression had the highest total effect on BMI, general health, and diabetes quality of life. Depression also had the highest direct effect on BMI and general health and the largest indirect effect on HbA1c, general health, and diabetes quality of life. Exercise and foot care also had the highest total effect and direct effect on BMI as depression does but in the opposite direction. The indirect effect of exercise and foot care on HbA1c and general health was also as large as depression with direction in the opposite. BMI had the largest total effect and direct effect on HbA1c. General health had the highest total effect and direct effect on diabetes quality of life.

The SEM model fits the data very well. The chi-square test (*χ*_9_^2^ = 8.529) for the goodness of fit has a *P* value of 0.482 with the other two goodness of fit indices of RMSEA = 0.000 (95% confidence interval: 0.000, 0.075) and CFI = 1.000.

## 4. Discussion

This study explored the associations between psychosocial and physiological variables and diabetes' indicators for Asians and Pacific Islanders with type 2 diabetes. The effect of health behavior on health outcomes is consistent with previous studies on type 2 diabetes [2–10]. The results from the SEM models indicated the positive effects of exercise and foot care on all health indicators. Regular exercise and foot care was with higher health behavior scores (good health behavior) related to lower HbA1c values and lower depression scores. For both diabetes quality of life and general health, the SEM models indicate that good health behaviors are associated with better diabetes quality of life and better general health.

The SEM model indicated a positive association between diet and exercise. Participants who were on diet were also more likely doing regular exercise and foot care. However, more depressed participants were less likely on diet and doing regular exercise and foot care. Diet had a direct association with diabetes quality of life, with those on diet having better quality of life. Regular exercise and foot care was associated with decreased BMI; however, increased depression level was associated with increased BMI. Higher BMI was associated with elevated HbA1c levels and worse general health. Elevated HbA1c levels also contributed to worse general health, and worsened general health would further decrease diabetes quality of life. Higher depression was also associated with worse general health and diabetes quality of life. 

The SEM models confirm the significant positive associations between health behavior and health indicators, reported in previous studies [2–10]. Our SEM models showed both the direct and indirect effects of health behavior and depression on diabetes patients' BMI, glycemic control, quality of life, and general health and highlighted the importance of exercise and depression on improving diabetes' patients' BMI, glycemic control, diabetes quality of life, and general health. 

Finally, the SEM models are used to disentangle the association between diet, exercise and foot care, depression, and health indicators such as BMI, HbA1c, general health, and diabetes quality of life. As the diabetes prevalence increases in Asians and Pacific Islanders than Caucasians, further research with more Asian and Pacific Islander subjects is needed to better understand the relationship between the psychosocial and physiological factors and health indicators.

The limitations of this study and analysis are that the SEM models based on the 207 Asians and Pacific Islanders with type 2 diabetes were from a cross-sectional data set. Thus, statistical significance shown in the SEM models may not necessarily indicate causality. Nevertheless, the SEM models provide some useful information that may lay the premise for future studies.

## 5. Conclusion

The SEM model was used to study the associations between psychosocial and physiological factors and diabetes' health indicators among Asians and Pacific Islanders. The results from the SEM model highlighted the importance of depression and exercise and foot care on improving diabetes patients' health indicators for Asians and Pacific Islanders. Future intervention-based studies on Asians and Pacific Islanders with type 2 diabetes need to focus on reducing patients' depression besides motivating them for regular exercise and foot care. 

## Figures and Tables

**Table 1:** Patients' demographic and clinical characteristics used in the SEM models.

Characteristics	*N *	%
Gender		
Female	113	54.6%
Male	94	45.4%
Marital status		
Single	26	12.9%
Married	141	69.8%
Separated/divorced/widowed	35	17.3%
Smoking status		
Smoker	20	10.0%
Nonsmoker	181	90.0%
Occupational status		
Professional, managerial	68	35.1%
Technical, clerical, and sales	34	17.5%
Service	21	10.8%
Other	71	36.6%
Education		
Less than 12th grade	19	9.5%
High school graduate	22	11.0%
Some college/associate	65	32.5%
Bachelor's degree	55	27.5%
Graduate school	39	19.5%
Income		
$24,999 or less	31	16.2%
$25,000–$49,999	43	22.5%
$45,000–$69,999	40	20.9%
$70,000 and above	77	40.3%

	Mean	Standard deviation

Age	57.4	10.9
BMI	32.2	7.3
Glycemic control (HbA1c)	8.0	1.6
Depression	10.1	8.9
Diabetes quality of life	73.6	14.2
General health	69.6	20.1
Diet	3.6	1.4
Exercise and foot care	3.2	1.5
Blood glucose and medication	4.7	1.7

**Table 2:** Pearson's correlation coefficients of variables used in the SEM.

Variables	BMI	Diet	Exercise and foot care	Blood glucose and medication	Depression	HbA1c	General health	Diabetes quality of life
BMI	1.00	−0.22	−0.49*	−0.15	0.41*	0.37*	−0.53*	−0.35*
Diet	−0.22	1.00	0.29*	0.19*	−0.20*	−0.19*	0.12	0.21*
Exercise and foot care	−0.49*	0.29*	1.00	0.17*	−0.14*	−0.09	0.23*	0.17*
Blood glucose and medication	−0.15	0.19*	0.17*	1.00	−0.06	0.04	0.05	0.03
Depression	0.41*	−0.20*	−0.14*	−0.06	1.00	0.21*	−0.62*	−0.49*
HbA1c	0.37*	−0.19*	−0.09	0.04	0.21*	1.00	−0.35*	−0.28*
General health	−0.53*	0.12	0.23*	0.05	−0.62*	−0.35*	1.00	0.58*
Diabetes quality of life	−0.35*	0.21*	0.17*	0.03	−0.49*	−0.28*	0.58*	1.00

*Denotes significant Pearson correlation coefficients with *P* value <0.05.

**Table 3:** Estimated unstandardized regression coefficients in the SEM.

SEM model	Estimate	S.E.	*P *
BMI ← exercise and foot care	−1.970	0.448	<0.001
BMI ← depression	0.327	0.085	<0.001
HbA1c ← BMI	0.077	0.021	<0.001
General health ← HbA1c	−2.025	0.795	0.011
General health ← depression	−1.069	0.150	<0.001
General health ← BMI	−0.688	0.252	0.006
Diabetes QOL ← general health	0.322	0.050	<0.001
Diabetes QOL ← depression	−0.292	0.114	0.011
Diabetes QOL ← diet	1.195	0.575	0.038

← Denotes the direct effect of the variable on the right-hand side to the variable on the left-hand side.

**Table 4:** Estimated correlations in the SEM.

SEM model	Estimate
Depression ↔ exercise and foot care	−0.139
Diet ↔ depression	−0.200
Diet ↔ exercise and foot care	0.293

**Table 5:** Standardized total effects, direct effects, and indirect effects in SEM.

	Exercise and foot care	Depression	BMI	HbA1c	Diet	General health
Total effects						
BMI	−0.40	0.40	0.00	0.00	0.00	0.00
HbA1c	−0.14	0.14	0.35	0.00	0.00	0.00
General health	0.12	−0.60	−0.31	−0.16	0.00	0.00
Diabetes quality of life	0.06	−0.46	−0.14	−0.07	0.12	0.45
Direct effects						
BMI	−0.40	0.40	0.00	0.00	0.00	0.00
HbA1c	0.00	0.00	0.35	0.00	0.00	0.00
General health	0.00	−0.48	−0.25	−0.16	0.00	0.00
Diabetes quality of life	0.00	−0.18	0.00	0.00	0.12	0.45
Indirect effects						
BMI	0.00	0.00	0.00	0.00	0.00	0.00
HbA1c	−0.14	0.14	0.00	0.00	0.00	0.00
General health	0.12	−0.12	−0.06	0.00	0.00	0.00
Diabetes quality of life	0.06	−0.27	−0.14	−0.07	0.00	0.00

## Abbreviations

API: Asians and Pacific Islanders
CFI: Comparative fit index
RMSEA: Room mean square error of approximation
SEM: Structural equation modelling.
